# A Case of Necrotic Metastases Imitating Hepatic Abscesses in a Patient With Multiple Primary Cancers

**DOI:** 10.7759/cureus.92466

**Published:** 2025-09-16

**Authors:** Abshar Khan, Cody King, Neerav B Patel, Aaryan Patel, Zaheer Irani, Joel Thompson

**Affiliations:** 1 Radiology, Lake Erie College of Osteopathic Medicine, Erie, USA; 2 Radiology, Rochester General Hospital, Rochester, USA; 3 Internal Medicine, Lake Erie College of Osteopathic Medicine, Erie, USA; 4 Physical Medicine and Rehabilitation, Lake Erie College of Osteopathic Medicine, Erie, USA

**Keywords:** adc mapping, ct, diagnostic radiology, dwi, hepatic abscess, malignancy, malignant liver abscess, mri, necrotic metastasis

## Abstract

This case report highlights the diagnostic challenges in a 72-year-old female with a complex medical history, including metastatic stage IV squamous cell carcinoma of the right lung, invasive ductal carcinoma of the left breast, and recent acute illnesses. Initially, imaging studies suggested metastatic disease in the liver, but subsequent findings raised suspicion for a hepatic abscess. However, further investigations ultimately confirmed necrotic metastatic disease rather than infection. This case underscores the importance of multidisciplinary collaboration and advanced imaging in differentiating metastatic disease from an infectious process.

## Introduction

Patients with metastatic cancer often present with diagnostic complexities, as imaging findings can mimic multiple etiologies, including infections in those who are immunocompromised due to ongoing chemotherapy. As one is of infectious etiology and another from a neoplastic process, their management is far from similar. The need for a concrete diagnosis is essential, and as these two pathologies can appear radiographically similar, potential misdiagnoses and delays in appropriate treatment may occur. Prior literature has documented cases where metastatic disease and infections were difficult to distinguish, emphasizing the need for histopathological confirmation [1]. Previous studies have noted that liver metastases, such as from neuroendocrine tumors and colorectal cancers, have mimicked abscesses due to necrotic changes or secondary infections. Such a study showed that a rich arterial supply of a neuroendocrine tumor metastasized to the liver displayed marked contrast enhancement. They may then develop central necrosis that appears radiographically similar to hepatic abscesses, with peripheral rim-like enhancement in the pyogenic phase [1,2]. Imaging modalities, such as diffusion weighted imaging (DWI) and apparent diffusion coefficient (ADC) mapping, can often aid in narrowing the differential but may not always be conclusive. This case emphasizes the critical role of imaging modalities, histopathological evaluation, and multidisciplinary tumor board discussions in establishing an accurate diagnosis and optimizing the management of this patient's care.

## Case presentation

A 72-year-old female with known metastatic stage IV squamous cell carcinoma of the right lung and invasive ductal carcinoma of the left breast presented to the emergency department with acute right upper quadrant pain, nausea, and vomiting. Her medical history was notable for a recent 19-day hospitalization for acute kidney injury (AKI), COVID-19 infection, pancytopenia secondary to recent chemotherapy requiring transfusion, and deep vein thrombosis (DVT) treated with Eliquis. Over a month prior to her admission, a computed tomography (CT) scan showed progression of lung cancer, with new and enlarging solid pulmonary nodules, an increase in size of a right adrenal mass, and a new liver lesion in segment 4A.

On the current admission, her vital signs showed an elevated blood pressure of 164/94 with the remaining vital signs within normal limits. Physical exam showed tenderness and guarding to palpation in the right upper quadrant and epigastric region. Laboratory evaluation upon admission revealed the following results (Table 1).

**Table 1:** Relevant patient lab results upon admission

Laboratory Test	Result	Reference Range
White Blood Cell Count (WBC)	16,600 /µL	5,000 – 10,000 /µL [3]
Hemoglobin	8.3 g/dL	12.0 – 16.0 g/dL [3]
Hematocrit	26%	37 – 47% [3]
Alkaline Phosphatase (ALP)	325 U/L	30 – 120 U/L [3]
Aspartate Aminotransferase (AST)	44 U/L	0 – 35 U/L [3]
Total Bilirubin	0.9 mg/dL	0.3 – 1 mg/dL [3]

**Figure 1:**
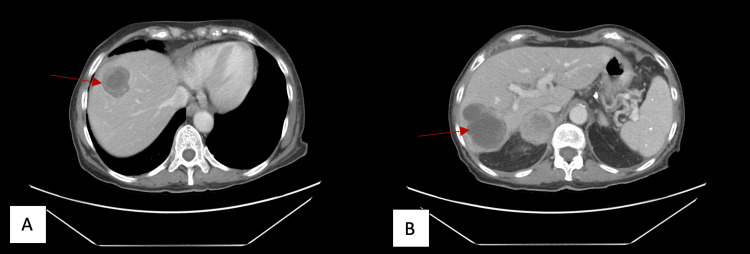
Axial CT abdomen and pelvis portal venous phase shows two hypoattenuating liver masses with rim enhancement and internal septations measuring 4.9 cm x 4.1 cm (A) and 6.7 cm x 5.6 cm (B) in the superior and inferior right hepatic lobes, respectively

The patient initially underwent imaging, including abdominal ultrasound and CT abdomen and pelvis. These studies reported findings concerning for acute cholecystitis, including gallstones, gallbladder distention, and gallbladder wall thickening. Additionally, a large heterogeneous mass was located within the gallbladder, measuring up to 8.8 cm. The patient was empirically started on piperacillin-tazobactam for suspected acute cholecystitis. Despite antibiotic therapy, her total bilirubin began to rise, and a hepatobiliary iminodiacetic acid (HIDA) scan showed no visualization of the gallbladder, supporting a diagnosis of acute cholecystitis. In regard to metastatic disease on the CT abdomen and pelvis, two hepatic lesions were slightly larger with decreased internal enhancement compared to a CT approximately six weeks prior. Figure 1 shows the axial CT abdomen and pelvis of our patient.

Magnetic resonance cholangiopancreatography (MRCP) was initially ordered to evaluate for choledocholithiasis, which was negative. Infectious disease consultants recommended aspiration and biopsy. Interventional radiology performed CT-guided drainage of the inferior hepatic lesion, and cloudy fluid was aspirated and sent for histopathological and microbiological analysis. Blood and aspirated fluid cultures were negative, possibly due to prior antibiotic therapy.

**Figure 2:**
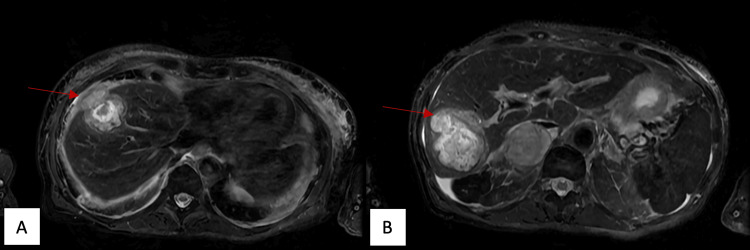
Fat-saturated T2 MRI images showing high internal T2 signal with mildly elevated peripheral T2 signal in the superior (A) and inferior (B) lesions

**Figure 3:**
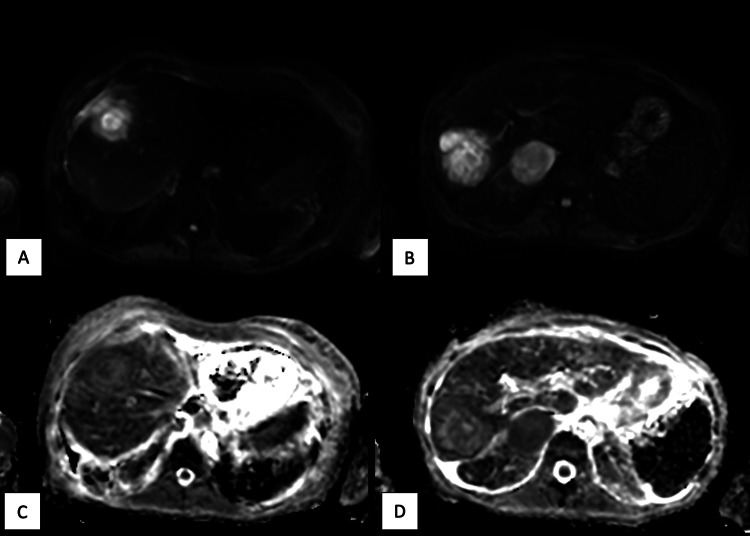
DWI MRI sequences of the superior (A) and inferior (B) lesions and ADC MRI sequences of the superior (C) and inferior (D) lesions showing internal hyperintensity on DWI with intermediate to low ADC values in the periphery of the lesions DWI: diffusion weighted imaging; ADC: apparent diffusion coefficient

**Figure 4:**
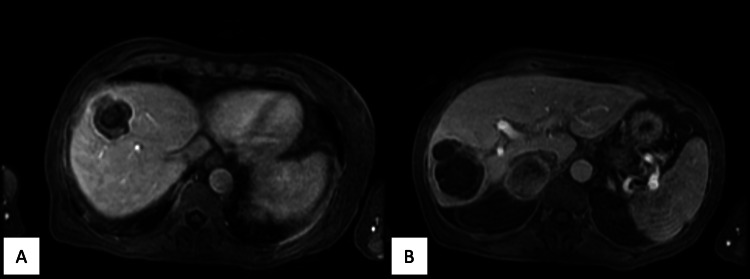
Axial T1 postcontrast late arterial phase MRI showing internal T1 hypointensity with rim and mild adjacent patchy parenchymal enhancement that persists through both phases of the superior (A) and inferior (B) lesions

Despite initial improvement, the patient developed recurrent fevers and rising white blood cell counts, prompting repeat imaging. A magnetic resonance imaging (MRI) study of the abdomen with and without contrast was ordered for further characterization of the hepatic lesions, which revealed an increasing size of the superior hepatic lesion that had not been previously drained, despite antibiotic administration. The differential diagnosis of necrotic metastases or abscesses was provided due to the lack of response to antibiotic treatment. This also allowed for further characterization of the previously visualized gallbladder lesion, which did not enhance and was favored to represent tumefactive sludge, blood products, or debris. However, MRI characteristics of the hepatic lesions began to raise suspicion for abscesses rather than metastatic disease. Figures 2-4 show the relevant characteristics of the hepatic lesions on MRI that led to the shift in differential diagnoses. 

Given these findings, interventional radiology attempted further drainage and biopsy.

The procedure revealed that the superior lesion could not be aspirated, raising suspicion for necrotic metastasis rather than an abscess. Additionally, its increasing size despite antibiotic administration led providers to believe her persistent leukocytosis was related to the persistence of a growing and decaying metastatic lesion. Due to the inability to aspirate, multiple core biopsies were taken from the superior lesion and sent for pathology and culture. Eventually, histopathological analysis confirmed metastatic squamous cell carcinoma within the superior hepatic lesion. Our patient’s intermittent fevers were ultimately attributed to her underlying malignancy rather than an infectious source. Unfortunately, her metastatic squamous cell carcinoma rapidly progressed over the next month, and the patient ultimately chose palliative care instead of further treatment.

## Discussion

This case underscores the complexity of diagnosing new hepatic lesions in patients with a history of metastatic cancer. New hepatic lesions on restaging CT imaging were presumed to represent metastatic disease. However, persistent low-grade fevers and the multidisciplinary input of radiology, infectious disease, and oncology teams shifted the diagnostic focus to an infectious etiology. The eventual identification of carcinoma within the hepatic lesions confirmed that these were, in fact, necrotic metastases, altering the management plan. This highlights the importance of considering necrotic tumor processes in immunocompromised oncology patients, as they can mimic abscesses on imaging.

Prior studies have documented the overlapping imaging features between metastatic disease and hepatic abscesses, emphasizing the potential for misdiagnosis and the necessity of histopathological confirmation. Hepatic metastases may develop necrosis or secondary infection, leading to overlapping imaging features with abscesses, including hypoattenuation on CT, peripheral rim enhancement, diffusion restriction, T1 hypointensity, and T2 hyperintensity [4,5]. The combination of CT and MRI findings has been shown to have a higher diagnostic accuracy than CT alone [4]. Studies have suggested that dynamic imaging may help differentiate between the two entities, with arterial phase patchy parenchymal enhancement and lack of peripheral enhancement washout on delayed images being more common in hepatic abscesses [4,6]. However, this unusual case of squamous cell metastases showed both of these characteristics. Prior studies have also shown that there can be significant overlap on DWI and ADC sequences depending on the degree of tumor necrosis and abscess maturation; however, the evaluation of the mass periphery on these sequences shows high diagnostic accuracy in differentiating between these entities at any stage [5,6]. While both pathologies can show peripheral DWI hyperintensity, abscesses have high peripheral ADC values due to T2 shine through, and metastatic disease reveals low peripheral ADC values due to restricted diffusion [6]. This is due to differences in viscosity, with the peripheral abscess being composed of increased extracellular fluid and inflammatory cells with a low viscosity, while the peripheral metastatic mass is highly cellular with a higher viscosity [6]. In this case, peripheral DWI hyperintensity and low peripheral ADC values were observed in both lesions, which could have favored the diagnosis of metastatic disease over abscesses, despite the many overlapping features. Although they may aid in the diagnostic workup, there is still variability in the ADC measurements between lesions of even the same histologic type, and there is no consensus about cutoff values in normal parenchyma, benign, and malignant lesions [7].

Misinterpretation of imaging findings can lead to delays in proper oncologic treatment or unnecessary prolonged antibiotic therapy. This case reinforces the importance of a comprehensive diagnostic approach that includes multidisciplinary collaboration and tissue sampling, when feasible, due to the complexity and degree of overlap between their imaging characteristics.

## Conclusions

Hepatic abscesses and necrotic metastases can appear similar on imaging, complicating the diagnosis, particularly in patients with a history of malignancy. This case shows how focusing on the mass periphery on DWI and ADC mapping may help differentiate between metastatic disease and hepatic abscesses when there are many overlapping imaging characteristics. As seen, peripheral DWI hyperintensity with low peripheral ADC increases the likelihood of necrotic metastasis in the differential, but imaging overlap still mandates tissue confirmation when feasible. Additionally, the procedural fact of non-aspirability supported malignancy in this case. This case demonstrated how neither single sign was fully determinative and highlights the importance of histopathological confirmation to ensure proper treatment strategies. Awareness of such diagnostic challenges can improve outcomes by preventing delays in appropriate treatment and ensuring that both infectious and oncologic causes are carefully considered in complex cancer patients.
